# Male and female mice show equal variability in food intake across 4-day spans that encompass estrous cycles

**DOI:** 10.1371/journal.pone.0218935

**Published:** 2019-07-15

**Authors:** Benjamin Smarr, Neil E. Rowland, Irving Zucker

**Affiliations:** 1 Department of Psychology, University of California, Berkeley, United States of America; 2 Department of Psychology, University of Florida, Gainesville, United States of America; 3 Department of Integrative Biology, University of California, Berkeley, United States of America; Kent State University, UNITED STATES

## Abstract

The exclusion of female rodents from biomedical research is well documented and persists in large part due to perceptions that ovulatory cycles render female traits more variable than those of males, and females must be tested at each of four stages of the estrous cycle to generate reliable data. These beliefs are not empirically based. The magnitude of trait variance associated with the estrous cycle may be sufficiently low and of little impact, or trait variability of males tested on 4 consecutive days may be as great as that of females over the 4 days of the estrous cycle. Here, we analyzed food intake data from mice in 4-day blocks, corresponding to the females’ 4-day estrous cycle in several schedules of food procurement or reward. Variance was compared within and across individual mice. In no instance did the overall variance differ by sex under any of the food reward schedules. This extends earlier observations of trait variability in body temperature and locomotor activity of mice and supports the claim that there is no empirical basis for excluding female rodents from biomedical research.

## Introduction

The exclusion of female rodents from biomedical research has been well documented [1–5]. Female rats and mice were omitted from experimental protocols because of presumed greater variability, attributed to hormonal changes associated with 4-day estrous cycles. Metanalyses in both mice[6] and rats[7], however, found that trait variability, tested without regard to stage of the estrous cycle, was not greater in females than males. Indeed, variability of core body temperature and locomotor activity was greater in male than female BALB/c mice within days, despite structured variance of estrous cycles across days in females. This suggests that study of female mice is particularly advantageous where observations are made over the course of several hours[8].

Many researchers still are prejudiced against female rodent models. Approximately two thirds of respondents in a 2017 survey of 1161 NIH study section members thought it important to consider sex as a biological variable in study design (i.e., include females), but others cited the need for increased funds if females were added to protocols to accommodate varying stages of the estrous cycle[9]. Residual prejudice against including females in rodent research remains entrenched in a segment of the scientific community. This, however, ignores the finding that there is no loss of statistical power to detect treatment effects in mixed-sex studies that do not require increases in sample size, as long as sex is used as an explicit factor and the effects on each sex are similar; only when treatment effects differ by sex must males and females be studied in separate groups and higher numbers[10].

Although trait variability correlated with stages of the 4-day rodent estrous cycle is well established for rats and mice[11,12], with one exception[8] we are unaware of studies that explicitly compare variability of behavior of males and females tested concurrently over the course of 4 consecutive days. It remains possible that males and females exhibit comparable trait variability or that females are more variable over the course of 4 consecutive days. We evaluated this conjecture by comparing food intake of mice tested during 4 consecutive days under different conditions of food procurement using operant behavior. The estrous cycle of mice is 4 days long, so in every test condition each female displayed the variability of its entire estrous cycle. Females were not staged, and there is no reason to assume inter-individual synchrony of estrous cycles. This allows a comparison between the variability of females and males across 4-day cycles. Our *de novo* analysis was based on previously published data[13,14], and an unpublished experiment conducted under the same conditions in the Florida laboratory, all with goals unrelated to those of the present study.

## Methods

House mice (*Mus musculus*) of the C57BL/6 (B6) strain were purchased from Envigo (Indianapolis, IN) at 3 months of age and were individually housed in vivaria maintained at 23–24°C and 40–70% relative humidity with 12L:12D light-dark cycles (lights on 0600–1800 h). During acclimation animals lived in polycarbonate cages with free access to Purina 5001 or Harlan 7912 standard chow pellets and water. The studies were carried out in strict accordance with the recommendations in the NRC Guide for the Care and Use of Laboratory Animals and the protocols were approved for each study by the University of Florida Animal Care and Use Committee. During experiments, animals were kept in individual behavior test chambers described in detail elsewhere [13,14]. All animals were sacrificed after experiments. Response devices and pellet deliveries were controlled by computers running Med PC-IV. Each daily session was 23 h long during which the number of responses and pellets delivered were acquired in time bins, every 5–15 min. In experiments 1 and 2 mice lived in operant chambers for 23 h per day and had four 40 min food opportunities starting at 1800, 2200, 0200 and 0600 h, during which a nose poke delivered a 20-mg grain-based food pellet according to the prevailing reinforcement schedule. In Experiment 3, food rewards could be earned throughout the 23 h test session. In all three experiments mice were removed from the test chamber each day and held in boxes without food or water for up to 1h while chambers were serviced.

### Experiment 1. Fixed interval (FI) experiment

Six male and 6 female mice lived in operant chambers for 23h /day; a cue light above the nose poke recess was illuminated for four 40 min food opportunities starting at 1800, 2200, 0200 and 0600 h during which time responses delivered a 20 mg food pellet according to the prevailing FI schedule (10, 20, 30 or 50 sec delays). Two nose pokes after the FI had elapsed since the previous pellet were required to obtain a food pellet. Responses before the end of each FI had no effect on pellet delivery. Females weighed ~15% less than males throughout the study.

### Experiment 2. Fixed unit price (FUP) experiment

Six male and 6 female mice lived in the operant chambers; nose pokes emitted while the recess was illuminated for 40 min at 1800, 2200, 0200 and 0600 h delivered a food pellet according to the prevailing FUP (or ratio) schedule. The number of nose poke responses required to deliver a pellet was increased in the sequence FUP 2, 5, 10, 25. After initial stabilization of intake at a fixed ratio two responses per pellet (FR2) for 2–3 days, an incrementing FUP series was initiated. The FUP progression of 2, 5, 10, 25 was employed for 4 days at each FUP for a total of 16 days. These data are from a previously unpublished study, but similar in procedure and results to Experiment 2.4 in ^13^.

### Experiment 3. FUP protocol with continuous access to rewards

Six male and 8 female mice initially weighed a mean of 19 g and 27 g, respectively. Completion of each nose poke delivered a single 20-mg pellet. Similar to experiment 2, data were collected in five contiguous 4-day phases (FUP2, FUP5, FUP10, FUP25, and FUP50) but in this study food could be earned at any time during the 23 h test sessions.

### Data analysis

Standard deviation was used in place of variance to reduce the effect of individual outlier events, and more closely approximate normal distributions. Data were analyzed in Matlab R2018a. Figures were arranged in Adobe Photoshop. All group analyses were carried out using N-way ANOVAs for standard deviation by block and sex. Post-hoc analyses of within-block differences were one-tailed t-tests, based on a default assumption tested of increased female variance relative to males. Statistics were Bonferroni corrected for multiple comparisons; all values reported are post-correction values.

## Results

### Experiment 1. FI schedule

Food intake was higher in males than females in all phases of the experiment. In both sexes, intake tended to plateau after 1 or 2 days at each new FI. At low costs (FI 10, 20), daily and individual variations reflected in the error bars of food intake were larger in females than males, possibly due to estrous cycles. However, no 4-day cycles of intake were evident in individual females other than cycles of intake linked to changes in FI [13]. Data were reanalyzed for effects of time and of sex on both intra-individual and inter-individual variability.

**Fig 1:**
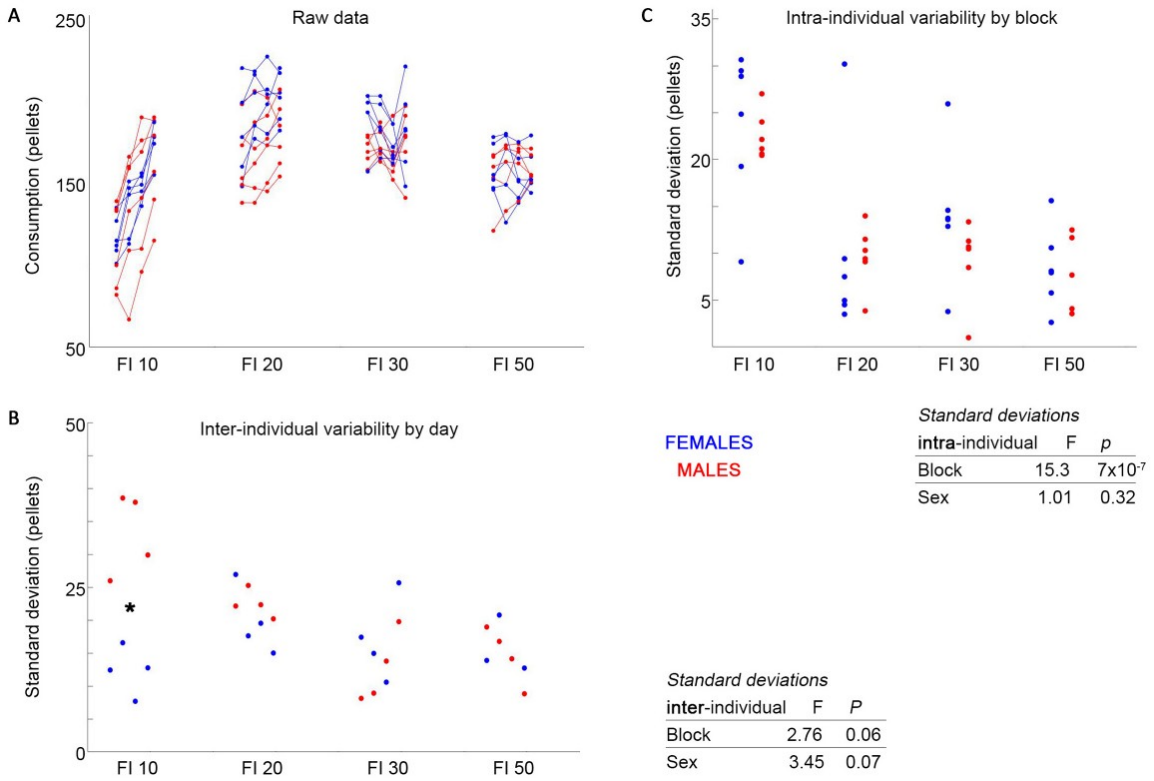
Female mice do not exceed males in variability, either within or across individuals. Previously reported feeding data (A) shows individuals given 4 days in each of 4 fixed-interval feeding regimens, with increasing intervals in each block. Intra-individual variability (B) and inter-individual variability (C) both show no significant increase in females over males. In one block for inter-individual variability, males significantly exceed females (C, *). Inter- and intra-individual variability significantly decreases with increasing cost.

Females did not show significantly greater intra-individual variability than males across blocks (Fig 1A). Within blocks, neither females nor males showed significantly higher intra-individual variability (Fig 1B). Females did not show higher inter-individual variability across blocks than males. In one block, males showed a significantly higher inter-individual variability than females (*p* = 0.03; first block; *, Fig 1C).

### Experiment 2.

**Fig 2:**
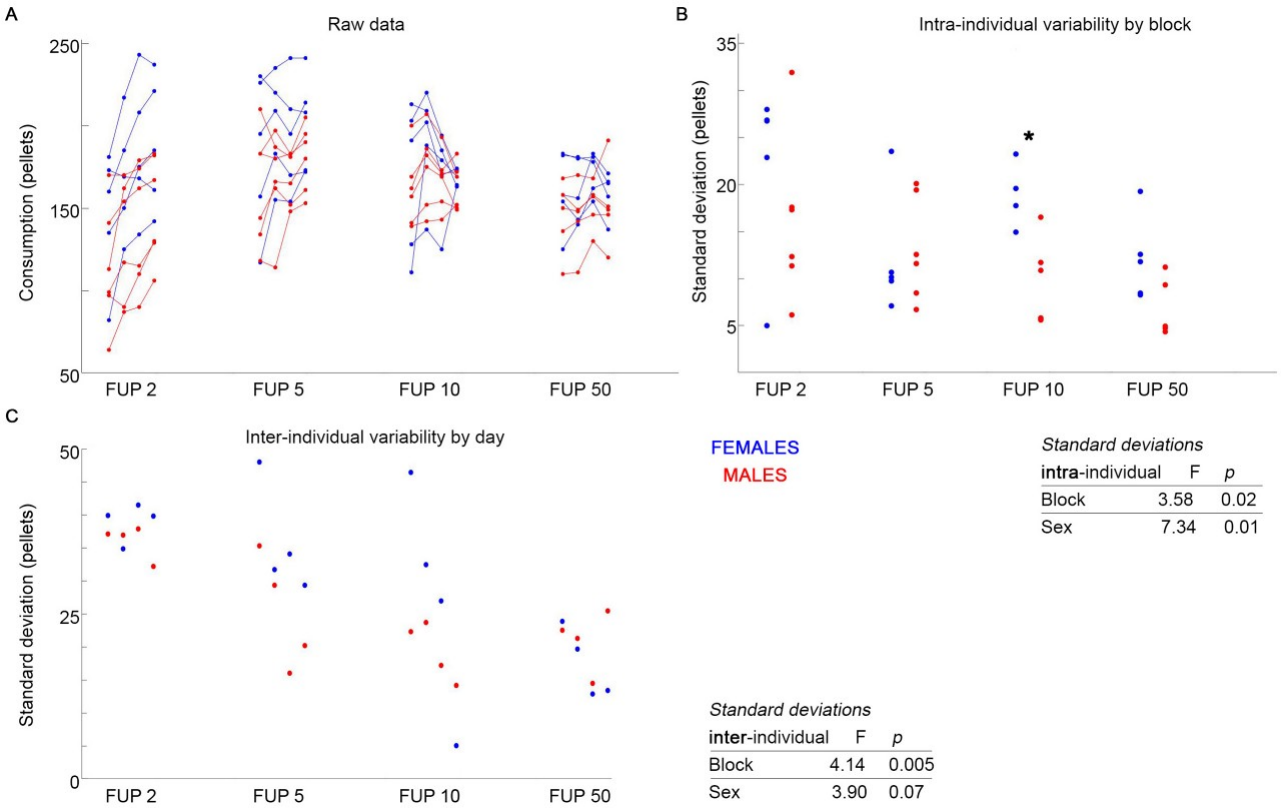
Under increasing cost of feeding, females exceed males in intra-individual variability, but not inter-individual variability. Previously reported feeding data (A) shows individuals given 4 days in each of 4 FUP feeding regimens, with increasing cost in each block. Females show significantly higher intra-individual variability (B) across blocks. Within blocks, females only significantly exceed males in block 3 (B, *). There is no sex effect in inter-individual variability (C). Inter- and intra-individual variability significantly decreases with increasing food cost.

Males weighed more than females and consumed more food during FUP2. One-way ANOVAs by sex were significant for females and males, with intakes at FUP2 and FUP5 > FUP10 and FUP 50, Fig 2A. No 4-day estrous-related cycles of intake in females were discernible other than those imposed by changes in FUP^13^.

For both males and females, and intra- and inter-individual variability decreased with increasing FUP (Fig 2B and 2C; F = 3.58, *p* = 0.02; F = 4.14, *p* = 0.005 respectively).

### Experiment 3. FUP with continuous food availability

Males and females showed different mean intake of food under 4-day blocks of increasing cost of food acquisition [13]. Data were reanalyzed for effects of time and sex on both intra-individual and inter-individual variability.

**Fig 3:**
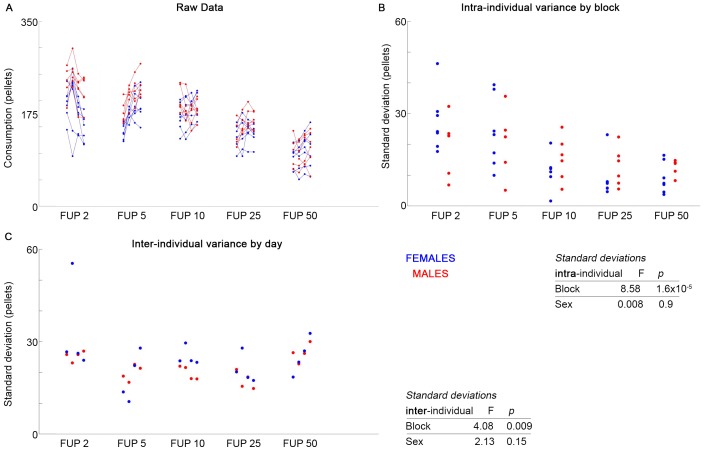
Under increasing cost of feeding, females do not exceed male variability. Previously reported feeding data (A) shows individuals given 4 days in each of 5 fixed-cost feeding regimens, with increasing cost in each block. Females show no significant increase in variance compared to males, whether for intra-individual (B) or inter-individual (C) variance, either across or within blocks.

Females did not show significantly greater intra- or inter-individual variability than males (Fig 3B and 3C; F = 0.008, *p* = 0.9; F = 2.13, *p* = 0.15, respectively) across or within blocks (post hoc analysis), though all mice showed significant decreases in intra- and inter-individual variability with increasing food cost (Fig 3B and 3C; F = 8.58, p = 1.6x10^-9^; F = 4.08, p = 0.009, respectively). The only significant effect of sex in the present analysis is the previously published one, that males show larger changes in food intake as a consequence of increasing cost^14^ (Fig 3A).

## Discussion

We found that males and females do not tend to show different levels of behavioral variance across any given 4 days. No significant effects emerged as a function of overall behavioral paradigms. Of the 26 possible post-hoc comparisons by sex for intra- and interindividual variance, two individual significant comparison differences were identified; one favored females, the other males, but none of these specific comparisons would normally be warranted, given the lack of overall experimental effect. Furthermore, in Experiment 1 variance of food intake in mice as a function of reward changed significantly more for males than females. Extrapolating from this finding into variance of measurements made without knowledge of physiological or environmental context suggests that males would show larger variance than females which is the opposite of the conclusion often drawn: that without knowledge of estrous context, females should be assumed to be more variable than males, and so excluded from experiments. In none of our testing conditions was there a significant effect of sex. Sex interacts with food cost and availability is specific ways but is not a substantial contributor to the variability of food intake, either within or across individual.

These analyses support enrollment of female mice, and incorporation of sex as a biological variable in preclinical research. Our findings do not substantiate claims that increased sample sizes and additional costs are incurred when females are deployed in experimental protocols.

Until recently, substantive data to support or refute the historical conjecture that supported exclusion of females from preclinical investigations were lacking. Two comprehensive analyses have demonstrated for mice [6] and rats [7] females tested at random stages of the estrous cycle are no more variable than males for numerous traits, consistent with genetic profiling arrays [15], and direct comparison of continuous locomotor activity and body temperature in mice [8]. The present study adds data from three studies of food intake behavior, and supports the same conclusion. An additional finding, also consistent with previous analyses from continuous data [8], is that in the absence of temporal or environmental context, males should be predicted to be more variable than females. Given that hormonal fluctuations impart structured variance across the estrous cycle, it remains unknown whether male variance can be accounted for by structure at a different timescale, or whether male variance–to the extent it is at least as great as female variance overall–is simply less structured. The source of variability in males corresponding to that found in the female estrous cycle remains to be identified.

We add two caveats. The first is that these findings are from a single but commonly-used mouse strain (B6). The extent to which the present findings may generalize to other mouse strains and other rodents is unknown. Second, these findings do not rule out the possibility of other estrogen-related effects. Indeed, using a similar operant protocol to Experiment 3, we reported that the elasticity of food demand (the relative decline of intake as FUP increased) was greater in female mice with knockout of the ER-α receptor [16]. This suggests that this receptor may improve resilience in the face of increasing food costs, a function that may facilitate the 2–3 fold increases of intake that occur in mice during the high energy demands of reproduction.

To establish that female mouse variability is greater than that of males one must concurrently compare trait variation over the course of the four-day estrous cycle with that of male counterparts. Such comparison are absent from the literature and the rare instances of implementation (present experiments and Smarr et al. [8]) fail to substantiate a sex difference in variability.

## Supporting information

S1 Table Data used for all analyses are available. (XLSX)

## References

[1] Berkley KJ. Vive la différence! Trends Neurosci. 1992 9;15(9):331–2. 1382330 10.1016/0166-2236(92)90048-d

[2] Mogil JS, Chanda ML. The case for the inclusion of female subjects in basic science studies of pain. Pain. 2005 9;117(1–2):1–5. 10.1016/j.pain.2005.06.020 16098670

[3] Beery AK, Zucker I. Sex bias in neuroscience and biomedical research. Neurosci Biobehav Rev. 2011 1;35(3):565–72. 10.1016/j.neubiorev.2010.07.002 20620164 PMC3008499

[4] Miller LR, Marks C, Becker JB, Hurn PD, Chen W-J, Woodruff T, et al Considering sex as a biological variable in preclinical research. FASEB J Off Publ Fed Am Soc Exp Biol. 2017;31(1):29–34. 10.1096/fj.201600781R PMC6191005 27682203

[5] Hughes RN. Sex still matters: has the prevalence of male-only studies of drug effects on rodent behaviour changed during the past decade? Behav Pharmacol. 2019 2;30(1):95–9. 10.1097/FBP.0000000000000410 29847339

[6] Prendergast BJ, Onishi KG, Zucker I. Female mice liberated for inclusion in neuroscience and biomedical research. Neurosci Biobehav Rev. 2014 3;40:1–5. 10.1016/j.neubiorev.2014.01.001 24456941

[7] Becker JB, Prendergast BJ, Liang JW. Female rats are not more variable than male rats: a meta-analysis of neuroscience studies. Biol Sex Differ. 2016;7:34 10.1186/s13293-016-0087-5 27468347 PMC4962440

[8] Smarr BL, Grant AD, Zucker I, Prendergast BJ, Kriegsfeld LJ. Sex differences in variability across timescales in BALB/c mice. Biol Sex Differ. 2017;8:7 10.1186/s13293-016-0125-3 28203366 PMC5301430

[9] Woitowich NC, Woodruff TK. Implementation of the NIH Sex-Inclusion Policy: Attitudes and Opinions of Study Section Members. J Womens Health 2002. 2019 1;28(1):9–16. 10.1089/jwh.2018.7396 30539677

[10] Beery AK. Inclusion of females does not increase variability in rodent research studies. Curr Opin Behav Sci. 2018 10;23:143–9. 10.1016/j.cobeha.2018.06.016 30560152 PMC6294461

[11] Quinlan MG, Duncan A, Loiselle C, Graffe N, Brake WG. Latent inhibition is affected by phase of estrous cycle in female rats. Brain Cogn. 2010 12;74(3):244–8. 10.1016/j.bandc.2010.08.003 20817338

[12] Datta S, Samanta D, Sinha P, Chakrabarti N. Gender features and estrous cycle variations of nocturnal behavior of mice after a single exposure to light at night. Physiol Behav. 2016 01;164(Pt A):113–22. 10.1016/j.physbeh.2016.05.049 27241632

[13] Rowland NE, Cervantez M, Robertson KL. Restricted temporal access to food and anorexia in mice: Microstructure of eating within feeding opportunities. Appetite. 2016 1 1;96:621–7. 10.1016/j.appet.2015.11.008 26589095

[14] Rowland NE, Minaya DM, Cervantez MR, Minervini V, Robertson KL. Differences in temporal aspects of food acquisition between rats and two strains of mice in a closed operant economy. Am J Physiol-Regul Integr Comp Physiol. 2015 5 20;309(2):R93–108. 10.1152/ajpregu.00085.2015 25994954

[15] Itoh Y, Arnold AP. Are females more variable than males in gene expression? Meta-analysis of microarray datasets. Biol Sex Differ. 2015;6:18 10.1186/s13293-015-0036-8 26557976 PMC4640155

[16] Minervini V, Rowland NE, Robertson KL, Foster TC. Role of estrogen receptor-α on food demand elasticity. J Exp Anal Behav. 2015 5;103(3):553–61. 10.1002/jeab.149 25869426 PMC4939438

